# Poor-Law Nursing in England: I. Sixty Years of Its History

**Published:** 1917-06-16

**Authors:** 


### THE MATRONS' AND SISTERS' DEPARTMENT.

### POOR-LAW NURSING IN ENGLAND.

### I.—Sixty Years of its History.

In considering the subject of Poor-Law nursing it is instructive to go back to the records of sixty years ago and describe the actual state of things as they existed in the workhouses of England before the advent of the trained nurse. The terrible state of the military hospitals in the Crimean War when Miss Nightingale first went out to nurse the sick and wounded there was equalled, if not surpassed, by the condition of the sick paupers in the workhouses at home. A report of the Poor-Law Board in 1866 on the London workhouses showed that their sick-wards were overcrowded, the beds far too close together, and "admirably contrived to induce bedsores"; that dirt, discomfort, and neglect abounded. The men wore the same shirts for seven weeks, and bed-clothing was sometimes not changed for months. The food was poor in quantity and quality, was cooked by paupers, and generally served quite cold.

The nursing—so called—was done by paupers, women who could neither read nor write. If medicine was ordered, it was poured into a gallipot by the nurse, "and the quantity judged according" was poured down the patient's throat. The medical officer's orders were not carried out properly, the sick and dying were often left without any attention at night, and were sometimes locked up in the wards till morning. Free fights frequently took place, and drunkenness, foul language, and vicious habits prevailed. This state of things was the same in the provinces, and especially in great cities like Liverpool.

The workhouse infirmary on Brownlow Hill was one of the largest in England, and contained over 1,200 beds. It was crowded with sick men, women, and children in every stage of illness and disease, in addition to the infirm and incurable. The respectable poor had such a horror of this parish hospital that they would endure any suffering or neglect in their own homes rather than enter its doors. In a seaport like Liverpool the women who went to the workhouse were of an exceptionally low class, and it was known that from these able-bodied paupers were selected the so-called nurses for the sick-wards.

In 1864 Mr. William Rathbone, a great philanthropist, who had already been the means of starting a system of district nursing among the poor of Liverpool, discovered how the infirmary at Brownlow Hill was detested by them. He investigated the matter, and on one occasion visited the wards himself with the Government inspector. The result was an anonymous offer to the Liverpool Vestry to pay for a certain number of trained nurses for three years if the Vestry would try the experiment of having them in the sick-wards. The offer was accepted, and Mr. Rathbone then wrote to Miss Nightingale, who had a few years earlier opened the training-school for nurses, called the Nightingale Home, at St. Thomas's Hospital, and asked her if she would supply suitable nurses. On May 16, 1865, Miss Agnes Jones, herself a Nightingale nurse, with twelve trained nurses and eighteen probationers, came to the Brownlow Hill Infirmary as lady superintendent, and took charge of all the male wards. The women's wards were left under the old conditions, but after two years the success of the experiment was so apparent and the good results so remarkable that the whole of the infirmary was put under Miss Jones and her nurses. The Vestry Board decided to charge the cost upon the rates, and later on Mr. Rathbone became a member of the Vestry, upon which he continued to serve for the rest of his life.

In studying the account given in the Memoirs of Agnes Jones, it is quite obvious that many of her difficulties at Brownlow Hill were caused by the attitude of the workhouse officials. The master, then called the governor, was at first inclined, and indeed promised, to help her; but differences soon arose, as they looked at things from a totally different standpoint. He thought her standard too high for the rough wards of a workhouse. What she, from her experience and hospital training, thought important, he considered fussy, trivial, or excessive.

Instead of the help and support she had hoped for came constant friction and opposition to any scheme either for reform in the wards or for the comfort of the patients. Miss Jones never overlooked the individual in the community. Workhouse officials are too apt to lump all classes together and treat them all alike, without any concern for their individual needs. In the case of the sick this is particularly unfortunate, and Miss Jones' experience of the attitude of the workhouse towards the "sick wards" has since been shared by many a superintendent nurse.

After three strenuous years Agnes Jones succumbed to typhus fever. But she will always be remembered as the pioneer of Poor-Law nursing. The work she accomplished in that short time was a tremendous one, and it was done under the most trying circumstances and depressing conditions. The nurses trained by her and fired by her example carried on the work, and Brownlow Hill Infirmary became a large training-school and is the Alma Mater of many a Poor-Law nurse to-day.

The example of Liverpool was speedily followed by other Unions all over the country, and the system of trained nursing was introduced into Poor-Law hospitals as quickly as suitable women could be trained for it. But a great deal of pauper help continued to be used in the wards, and it was not until thirty years later, in 1897, that an Act was passed forbidding paupers to act as nurses in workhouse infirmaries.

In 1867 an Act came into force which made it possible to separate infirmaries from the workhouse. All the Unions and parishes in London were formed, under this Act, into one district, called "The Metropolitan Asylums District," for the insane and for fever and smallpox cases, which till then had been treated in workhouses. In London separate infirmaries were built for all non-infectious sick and placed under medical superintendents. Unfortunately, this was not made compulsory, and therefore in the provinces many of the infirmaries remained under the authority of the workhouse.

The result of this is that there are now three grades or classes of Poor-Law hospitals, a fact not generally recognised by the lay mind.

1. Infirmaries which are quite separate and are often some distance from the workhouse. These training schools are under the control of a medical superintendent, and have a trained matron over the nursing and domestic staff.

2. Infirmaries which are separate buildings, but rank as part of the workhouse and are still under the control of the master. These are also large training-schools and the nurses are under the lady superintendent, as she is usually called, to distinguish her from the matron of the workhouse, who, in these infirmaries, does not visit or interfere in any way in the wards or with the work and conduct of the staff.

3. The sick wards of the workhouse. These are usually in a separate wing; or in a small adjoining building, in charge of a superintendent nurse, but under the control of the workhouse master and matron in all matters not actually concerned with the treatment of the sick.

For these the superintendent nurse is responsible to the medical officer, who is non-resident. These small infirmaries stand quite outside, and can never be treated as training-schools. The drawbacks and anomalies of this three-grade system and the difficulties connected with it will be shown in another article.

